# A Mnemonic for Effective “Lesss PAINFUL” Pre-Rounding

**DOI:** 10.7759/cureus.75393

**Published:** 2024-12-09

**Authors:** Michael C Larson, Akash Pathak, Ashita Tanwar, Daniel Linfesty, Jacob Lipovac, Katherine Liu

**Affiliations:** 1 Radiology, University of California Davis School of Medicine, Sacramento, USA; 2 Neurology, California Northstate University College of Medicine, Elk Grove, USA; 3 Medical School, California Northstate University College of Medicine, Elk Grove, USA; 4 Internal Medicine/Pediatrics, University of California Davis Health Medical Group, Sacramento, USA; 5 Medicine School, University of California Davis School of Medicine, Sacramento, USA

**Keywords:** medical education, medical mnemonic, medical skills, mnemonic, pre-rounding, rounding

## Abstract

Due to much of medical training being inpatient centered, medical trainees generally do more pre-rounding on a per-patient basis than they do complete histories and physical exams (H&Ps). However, formal training often overlooks pre-rounding as a critical aspect of medical education and patient care, with at least 10 times more publications on H&Ps than on any other aspect of rounding over the past half-century. To address this critical gap in medical education, we introduce the “Lesss PAINFUL” pre-rounding mnemonic, emphasizing the importance of efficient pre-rounding for medical students or other trainees. The mnemonic guides trainees through *P*rogress, *P*resenting symptoms, *P*hysical, *A*utonomy*, A*mbulation, *I*ns and Outs, *N*ursing, *N*eeds before discharge, and *F*ollow-*U*p *L*abs and imaging, providing a systematic framework to ease the burden of learning pre-rounding. The extra "s" in “Lesss” is to emphasize that each letter in the PAINFUL mnemonic represents multiple aspects per acronym letter. This mnemonic has substantial potential to streamline both medical training and inpatient care by serving as an effective internal checklist to ensure a thorough evaluation by the learner, thereby contributing to the overall quality of care for hospitalized patients. As medical education evolves, incorporating mnemonic-based strategies like "Lesss PAINFUL" holds promise for enriching and solidifying the skills of future healthcare practitioners.

## Introduction

Rounding is the act of a medical team reviewing the pertinent details and treatment plan for inpatients under the care of the team. Pre-rounding encompasses all the data gathering and planning about patients, typically by one or two trainees per patient, before rounding as the entire team when medical decisions are made. Depending on when the full team rounding starts, the number of patients needed to be seen, and the efficiency of the trainee, pre-rounding may start at 4 AM or earlier and typically involves waking up the patient for a brief interview and physical exam, which can be a mentally painful experience for trainees and their patients.

Pre-rounding on hospitalized patients is critical for efficient care by medical students, residents, advanced practice providers, and other allied health professionals. However, while medical students are often well-instructed in the fundamentals of history-taking and physical examinations, they often receive limited guidance on the essential skill of pre-rounding. Though hallway presentations and progress note updates represent a sizable portion of their clerkship experience, formal training for these scenarios is often minimal and inadequate [1]. Rather, pre-rounding is simply taught as the work done to draft a "SOAP note" and then a description of the SOAP acronym: Subjective, Objective, Assessment, Plan, and a brief description of what goes into each section of a SOAP note. As a result, medical students rely primarily on informal instruction from senior colleagues to develop this skill [2].

An average inpatient’s length of stay can be significantly reduced by implementing effective rounding tools [3]. Pre-rounding allows those on a team without final authority (such as trainees and midlevel providers) time to synthesize clinical information and form an accurate assessment before formal rounding with the team. By addressing key aspects of management before formal rounds, such providers can streamline care and focus on high-yield findings. An analysis of hospital data reveals that a proactive approach to systematic rounding has the potential to impact patient outcomes [4], making pre-rounding a critical skill for trainees to master.

There are many commonly-taught acronyms for general medical (e.g. MUDPILES (methanol, uremia, diabetic ketoacidosis, propylene glycol, isoniazid, iron, ; lactic acidosis, ethylene glycol, salicylates) anionic gap metabolic acidosis causes mnemonic, or SIGECAPS (sleep, interest, guilt, energy, concentration, appetite, psychomotor, suicide) depression symptoms mnemonic), or specialty-specific acronyms (e.g. FEGNOMASHIC (fibrous dysplasia or fibrous cortical defect, enchondroma or eosinophilic granuloma, giant cell tumor or geode, non-ossifying fibroma, osteoblastoma, metastasis(es)/myeloma, aneurysmal bone cyst, simple (unicameral) bone cyst, hyperparathyroidism (brown tumor), infection (osteomyelitis) or infarction (bone infarction) or intraosseous lipoma, chondroblastoma or chondromyxoid fibroma) lucent lesion differential diagnoses in musculoskeletal radiology).

As pre-rounding is a crucial skill for medical students and interns that plays a significant role in patient care, the authors aimed to develop a mnemonic that would assist trainees in conducting thorough, efficient, and consistent pre-rounding evaluations. This mnemonic has not been scientifically validated but is meant to serve as a memory aid, with future work needed to validate it before its use can be claimed to improve trainee confidence, efficiency, or any aspect of clinical care, none of which is claimed in this technical report.

## Technical report

The need for work on pre-rounding

**Figure 1:**
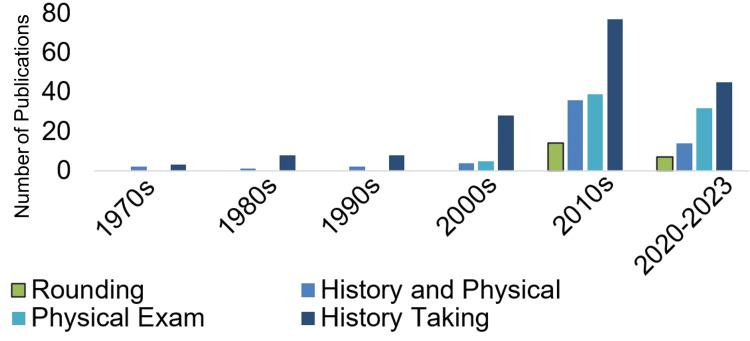
PubMed search results showing the relative lack of emphasis on rounding in medical education. Evaluation of the number of publications over time of “medical student” and various clinical skills, including “physical exam,” “history taking,” “history and physical”, and “rounding” highlighting the clear emphasis on history taking and physical exams in medical student education over hospital rounding.

To validate the need for a pre-rounding mnemonic, the National Institutes of Health's PubMed database was used to evaluate literature on medical student training. A query was done of PubMed for articles with the keywords "medical student" and the "AND" linker with either "physical exam," "history and physical," "history taking," or "rounding." No results were present with "medical student" AND "pre-rounding" as keyword combinations. The results of the keyword searches (done till December 8, 2023) are given in Figure 1.

The “Lesss PAINFUL” mnemonic

Nearly a decade ago, the senior author of this technical report thought it was a pain to have to start pre-rounding at or before 5 AM on certain rotations, and came up with a partial mnemonic that helped him through the internship year. The initial acronym mnemonic was PAIN: Progress/Physical, Ambulation/deep vein thrombosis (DVT), and other prophylaxis, Ins/Outs, Needs before discharge. As there was still a clear deficit in work done on pre-rounding as shown in Figure 1, the nascent mnemonic the lead author had mentally relied upon as an intern was further developed and refined by the other authors. The final mnemonic is described below. The extra ‘s’ in “Lesss” is to emphasize that each letter in the PAINFUL mnemonic represents multiple aspects per acronym letter.

P - Progress, Presenting Symptoms, and Focused Physical

‘P’ represents Progress, Presenting symptoms, and a focused Physical examination. Users of this mnemonic should begin with the end in mind, assessing progress toward the goal(s) of care. The medical student or intern needs to make sure to assess how the patient is feeling, check on changes in presenting symptoms compared to the previous day, and evaluate the progress of other patient-centered reason(s) for the hospitalization. Additionally, the symptom management plan has to be reviewed; Is it time to adjust the dose, frequency, and route of treatments? How often did the patient need PRN (pro re nata) medications? Lastly, perform a focused physical examination.

A - Autonomy and Ambulation

‘A’ emphasizes Autonomy and Ambulation. Vital signs are a measure of the body’s autonomous function. Assessing current vitals is as important as assessing trends over the hospitalization for additional insights. The medical student/intern should further evaluate patient autonomy factors such as precautions, restrictions, and other extrinsic or intrinsic limitations of activities of daily living (ADLs). One could even consider adding "ADLs" if regularly involved with geriatric care to the 'A' part of this mnemonic. Ensure the balance between patient autonomy and patient safety is continually re-evaluated through appropriate physical, occupational, or speech therapy consultations and prescribed precautions. Additionally, since the detrimental effects of immobility are highly prevalent in hospital care, ongoing physical movement should be encouraged as tolerated to promote overall wellness and deep vein thrombosis prophylaxis [5]. The student/intern should carefully assess safety with ambulation, including room safety, fall risk, airborne precautions, etc. Finally, in the critical care setting where patients’ organs may not be functioning autonomously, 'A' could be expanded to include evaluation of the Airway/Arterial lines and other devices, Assessments by organ system, and Antimicrobials appropriateness.

I - Ins and Outs

‘I’ stands for Ins and outs. All input through IVs (or ports or IOs) infusions and oral (or other enteric tube) intake should be evaluated and the patient should be inquired about any issues related to intake such as dysphagia, nausea, etc. Also, the medical student/intern should remember to review the IV fluids plan. Any upcoming procedures or other developments that may change the patient’s intake and discuss such with the patient should be definitely noted. Lastly, inquiry should be done about bowel and urine output and output from drains or other sources; documentation should be done if drains are flushed, stripped, or potentially no longer needed, but a drain should never be pulled if untrained in doing so.

N - Nursing and Needs for Discharge

‘N’ is a reminder for Nursing and Needs for discharge. Nurses and other providers involved in the patient’s care should be consulted to better understand overnight events. Consulting the rest of the team may provide details that are not immediately obvious or weren't thoroughly documented. Also, specific patient needs or other nuances before discharge should be addressed, such as wound care, goal-of-care discussions, transitioning to oral medications, any new home or care facility requirements, etc.

FUL - Follow-Up Labs and Imaging

‘FUL’ represents Follow-Up Labs and imaging. Results from any labs and imaging tests ordered should be reviewed, to ensure their completion. If needed, a radiologist or pathologist should be called for clarification. The test results should be discussed briefly with the patient, if appropriate. Finally, standing orders that are noncontributory to patient care should be canceled in many instances such as daily blood draws or daily chest radiographs.

The information obtained using the “Lesss PAINFUL” mnemonic can be seamlessly integrated into portions of a SOAP note or consolidated into a brief presentation during morning rounds. Medical students and interns can use this systematic approach for smoother preparation for rounds.

Using the Lesss PAINFUL mnemonic

Below is a hypothetical example of the “Lesss PAINFUL” mnemonic used by an intern, making special note of the ideal memory triggers from the mnemonic.

The intern knocks and enters the room of Mrs. Smith, an elderly lady admitted for heart failure. The intern considers how painful it is waking up the patient at such an early hour, triggering their memory of the “Lesss PAINFUL” mnemonic.

P for progress, presenting symptom, & physical: “Good morning, Mrs. Smith, how are you feeling today? Were you able to sleep better?” The intern takes note of only three pillows on her gurney and documents the pulse oximeter reading in the low 90s but slowly rising. Mrs. Smith awakens and groggily responds. The intern remembers that Mrs. Smith’s shortness of breath prompted her to first seek medical care and asks, “How is your breathing?” Moving on to the physical exam, the intern politely asks, “Do you mind if I loosen these calf compressors to see if the swelling has gone down in your legs?” The focused physical exam shows ongoing signs of heart failure, but significant improvements from the day before. The intern comments, “I saw in the chart that the cardiologist came by yesterday. It sounds like we still need to get your medications adjusted before we send you home, but we can discuss this more later.”

A for autonomy/ambulation: Finishing the exam, the intern says to Mrs. Smith, “Your lungs sound a little better and the swelling in your legs has gone down. It looks like your vital signs have been improving since you’ve been here. Were you able to make it on a walk with some help around the nursing station yesterday like we talked about?” The intern also glances around to ensure that ambulation is safe and moves the patient’s walker from across the room, closer to the bed as a subtle hint. Wanting to respect autonomy but balance safety, the intern thinks keeping the nasal cannula and fall precautions on is still prudent at this point but will reassess tomorrow before bringing it up at rounds.

I for Ins and Outs: The intern checks the chart for any IV or oral medications or documented oral intake while asking, “Are you able to eat more now that your shortness of breath has improved? Do you have any other concerns about eating and drinking or are you having any nausea or vomiting?” While Mrs. Smith indicates that oral intake hasn’t been an issue for her, the intern notices that there are two IVs still in place, scribbling “D/C 1x IV?” as a reminder to discuss this at rounds, and then moves on to output. “Are the water pills still tolerable? It looks like you’ve had a lot of urine output, but we expect that as part of the treatment process. Are you having any issues passing stool?”

N is for nursing and needs for discharge: The intern didn’t hear about anything happening at sign-out from the overnight team but will double check with the nurse to be sure. Besides the pending medication adjustment, the intern notes no other specific discharge needs.

FUL for Follow-Up Labs & Imaging: “Well, I’ll let you get back to sleep now Mrs. Smith. We can talk more about your echocardiogr, er, the ultrasound of your heart once the whole team is here, okay?” The intern then pulls up Mrs. Smith’s chart in the hallway computer with the updated labs and echocardiogram report, taking note to ask if the team really needs standing radiographs and blood draws now that things have normalized, and double checking with the nurse walking by that no significant events occurred overnight. The intern begins to draft the electronic note, jotting down a few highlights for a SOAP presentation and smiles about how pre-rounding today was a lot “Lesss PAINFUL” than it could have been.

## Discussion

The utilization of the “Lesss PAINFUL” mnemonic is intended to contribute both to the ease of learning pre-rounding techniques and hopefully provide more comprehensive patient care through intentional redundancy, though such a benefit would need to be proved with a formal trial that is beyond the scope of this technical report. A structured approach to pre-rounding helps trainees gather essential information, address patient needs, and ensure efficient patient management. Like other methodical mental and physical safety checklist methods in current literature, this mnemonic system has the potential to enhance patient outcomes and improve the overall quality of care [6]. Further, if the “Lesss PAINFUL” mnemonic is used as a literal checklist, similar to the well-known SOAP or OPQRST (Onset , Provocation/palliation, Quality, Region/Radiation, Severity, and Time) mnemonics, it has additional potential to enhance patient safety through improved clinical documentation, contributing to improved patient outcomes [7,8].

Potential limitations to this mnemonic include the trainee needing to memorize multiple aspects per letter of PAINFUL, e.g. P standing for “Progress, Presenting symptom & a focused Physical,” and I standing for “Ins/Outs,” which also includes multiple components since there are at least two common ways for input (PO and IV) and potentially more than just two means of output depending on patient factors. However, given that this mnemonic is intended to introduce and provide trainees with an organized method for pre-rounding, its value lies more in its ability to offer appropriate memory triggers in the process of information gathering. Since the components of PAINFUL are commonly encountered in the clinical setting, with practice, trainees will naturally begin to build associations with the mnemonic and further organize their pre-rounding. Without disrespect for the memorability of the CHADS2 (Congestive Heart Failure, Hypertension, Age ≥75 years, Diabetes mellitus, and Stroke) numeronym (an acronym with numbers) or subsequent work on the CHA2DS2-VASc (Congestive heart failure, Hypertension, Age ≥75 years (doubled), Diabetes mellitus, prior Stroke or TIA or thromboembolism (doubled), Vascular disease, Age 65 to 74 years, Sex category) numeronym highly-validated clinical calculator, an acronym is felt by the authors to be more easily memorized and more timeless than a numeronym should it be modified for ICU or other clinical scenarios.

A clear limitation to this mnemonic is its early stage of use, not having been formally validated in any way to improve trainee confidence, hasten the speed of pre-rounding, or any improvement in patient outcomes. Despite this limitation, which could also be said of MUDPILES and other educational mnemonics, it does address a gap perceived by the authors and colleagues in teaching pre-rounding skills in a memorable way. Future work addressing this could include direct surveys to medical students after learning this tool, or indirectly by comparison of faculty evaluation of students who have used this mnemonic versus the current standard figure-pre-rounding-out-on-your-own approach. Implementation of a formal medical record checklist spelling out “PAINFUL” in the chart may not be ideal when patients or their caregivers rightfully have access to such documentation, much like many are taught to not use the acronym “COW” for computer on wheels due to potentially inadvertently offend patients or others. Despite these limits, this mnemonic system has strong potential to help with a vital aspect of medical training.

Though often overlooked and undervalued, pre-rounding serves a critical role in medical student education, facilitating the students’ transition from data gatherer and reporter to the decision-making and analytic mindset of a clinician. Mnemonics are often invaluable teaching tools for medical students, offering a structured and memorable framework for routine tasks [9]. Such frameworks have been shown to enhance the knowledge and confidence level of providers, and further improve the speed and efficiency of patient care delivery and overall task completion [10,11]. The “Lesss PAINFUL” mnemonic aims to guide medical students toward more comprehensive and systematic pre-rounding, and hopefully can also provide a framework to independently arrive at an assessment and plan [12]. As medical education continues to evolve, the integration of updated mnemonic-based strategies such as “Lesss PAINFUL” into training curricula holds promise for enhancing and solidifying the practical skill set of future healthcare practitioners and enriching overall patient care.

## Conclusions

Efficient and thorough pre-rounding is a critical component of patient care and safety. The "Less PAINFUL" mnemonic offers medical trainees a systematic approach to pre-rounding, ensuring they address key patient concerns, survey all pertinent information, and contribute to the development of an accurate and holistic plan for patient care. While this mnemonic has not been formally validated, it holds the potential to assist trainees in improving patient care. Incorporating this approach into their training may help medical students become more confident and effective healthcare providers, ultimately benefiting their current and future patients.
